# Dynamic properties of excitons in ZnO/AlGaN/GaN hybrid nanostructures

**DOI:** 10.1038/srep07889

**Published:** 2015-01-20

**Authors:** Mathias Forsberg, Carl Hemmingsson, Hiroshi Amano, Galia Pozina

**Affiliations:** 1 Department of Physics, Chemistry and Biology (IFM), Linköping University, S-581 83 Linköping, Sweden; 2 Department of Electrical Engineering and Computer Science, Nagoya University, Chikusa-ku, Nagoya, 464-8603, Japan

## Abstract

Hybrid samples based on ZnO colloidal nanocrystals (NCs) deposited on AlGaN/GaN quantum well (QW) structures with different top barrier thickness *d* = 3, 6 and 9 nm are studied by time-resolved photoluminescence. Thermal behavior of the QW exciton lifetime in the hybrids and in the bare QW structures has been compared and it has been found that the QW exciton recombination rate increases in the hybrid having *d* = 3 nm and decreases in the hybrid with *d* = 6 nm, while no change has been observed for the structure with *d* = 9 nm. It is suggested that non-radiative resonance energy transfer from the QW excitons to the ZnO NCs and a variation of the surface potential can both influence the QW exciton lifetime in the hybrids.

III-nitride semiconductor/organic polymer hybrid heterostructures combine advantages of epitaxially grown semiconductor quantum wells (QW) with inexpensive polymers having efficient luminescence in the visible region^1^. Such hybrid micro-structured light emitting diodes (LED) are promising for fabrication of low-cost and highly efficient microlight sources that can be used in full-color displays, imaging systems, miniature chemical and biological sensors. In typical polyfluorene/GaN-based LED hybrids, UV emission from a GaN heterostructure down converts to the organic polymer fluorescence in the visible region via a radiative energy transfer. Overlapping between the UV luminescence and the polyfluorene absorption is required for the operation of these hybrids. Today, a novel class of hybrid structures is suggested, in which a non-radiative (Förster) resonant energy transfer (NRET) from excitation generated in inorganic QWs to excitons in organic films can be utilized^2^,^3^. Such LEDs might be considerably more efficient than their radiative energy transfer analogues^4^. In addition to the necessity of a significant spectral overlap between the QW emission and the polymer absorption spectrum, these devices require that the two materials are placed in a close interaction distance of a few nm. The bottleneck is that the operation lifetime of organic/semiconductor hybrid LED structures is limited by degradation of polyfluorenes. Using colloidal semiconductor nanocrystals (NCs) instead of polymers can significantly improve the lifetime of such devices^5^,^6^,^7^. In addition to superior luminescence properties, relatively low cost and chemical stability, the spectral tunability can be achieved by changing the particle chemistry and size. The efficiency of non-radiative resonance energy transfer is typically determined using transient photoluminescence (PL) measurements from the quenching of the QW exciton lifetime in the presence of acceptor material (i.e. colloidal NCs or polyfluorene)^4^,^5^,^6^. It might be correct in assumption that NRET is the only additional recombination channel appearing in hybrids compared to the bare QW structure. However, other factors can play also a significant role. For example, surface potential effects have to be considered when non-radiative resonant energy transfer is measured using dynamic properties of the QW excitons.

Thus, in this work we have studied and discussed the possibility of NRET in hybrid structures fabricated using ZnO NCs films coated on the top of the AlGaN/GaN QWs samples. ZnO NCs satisfies the requirement of absorption overlapping with GaN emission (a room temperature band gap energy is 3.3 and 3.4 eV for ZnO and GaN, respectively). Dynamic properties of QW excitons in the hybrids and in the bare QW samples are analyzed in dependence on the QWs cap layer thickness.

## Results

**Figure 1:**
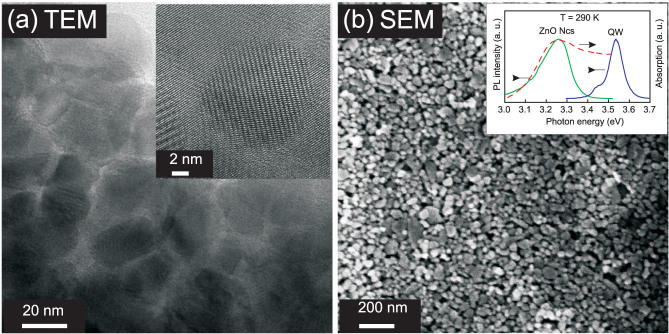
(a) TEM image of the ZnO NCs dispersed in ethanol. The insert shows one particle at high resolution. (b) SEM top view of the hybrid structure. Room temperature PL and absorption spectrum for ZnO NCs together with the AlGaN/GaN QW emission are shown in the insert.

Transmission electron microscopy (TEM) measurements has been performed to confirm the quality of the ZnO NCs. TEM image in Fig. 1(a) shows that ZnO NCs have a rather spherical shape and a single crystalline quality as it follows from the insert illustrating one particle with a ~25 nm diameter, where a lattice fringes can be resolved. Fig. 1(b) demonstrates an scanning electron microscopy (SEM) image of the ZnO NCs film, which covers rather uniformly the surface of the QW sample. As shown in the insert of Fig. 1 (b), a room temperature absorption spectrum of the ZnO NCs overlaps with the emission from the QW structure, thus satisfying NRET condition. In general, time-resolved photoluminescence (TRPL) properties of ZnO NCs were similar to those obtained previously for the ZnO nanoparticles of similar size^8^.

**Figure 2:**
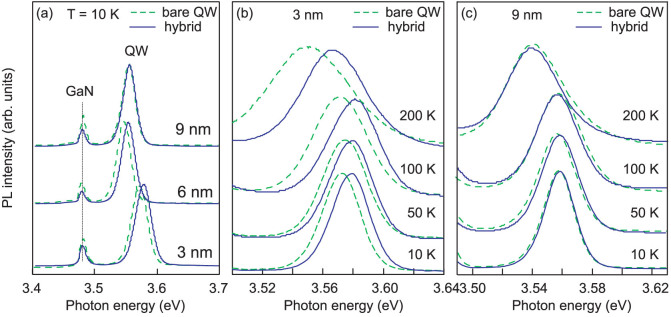
(a) Low-temperature PL spectra for the bare QW structures (dashed lines) and for samples coated with ZnO NCs film (solid lines). The capl layer thickness is indicated for each spectrum. (b) and (c) QW-related emission at several temperatures for coated (solid lines) and uncoated (dashed lines) structures with the cap layer thickness of 3 nm and 9 nm, respectively.

**Figure 3:**
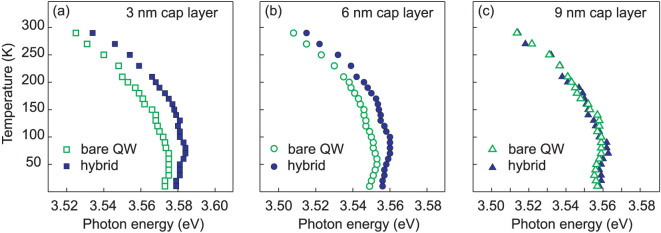
Temperature dependence of the QW PL peak position measured in the bare QW structures (open signs) and in the hybrids (solid signes) with the top layer thickness of (a) *d* = 3 nm, (b) *d* = 6 nm and (c) *d* = 9 nm.

PL spectra in the UV region measured at 10 K are presented in Fig. 2(a) for the bare QW structures (dashed lines) and for the hybrids (solid lines) with three different cap layer thicknesses. The main PL line corresponds to the QW exciton emission at ~3.57 eV and ~3.54 eV for the samples with 3 nm and 6 or 9 nm top layers, respectively. A weaker PL peak at ~3.48 eV corresponds to the GaN buffer layer. There is no difference in the spectrum shape and in the exciton peak energy between the bare QW sample and hybrid with the thickest cap layer (9 nm), while for the samples with 3 and 6 nm cap layer, the QW exciton position is shifted to higher energies (up to 20 meV) in the case of hybrid structures. The shift is present even at elevated temperatures for the hybrids with thinner cap layers in contrast to the hybrid with a 9 nm-thick spacer as illustrated in Fig. 2(b) and (c). In more details, temperature dependence of the exciton peak position is plotted in Fig. 3 for the bare QW samples (open signs) and for hybrids (solid signs). A slightly non-monotonic behavior with increasing temperature (so called S-shape) of the exciton peak position caused by thermal delocalization of excitons is observed in both bare QWs and hybrid structures. The maximal localization energy is estimated to ~8–10 meV at 90 K when the QW PL line is the most shifted to the higher energy region. As abovementioned, a change of the QW exciton energy in hybrids compared to the bare QW samples was found only in structures with 3 and 6 nm cap layers, see Fig 3(a) and (b), while no difference in the QW line position before and after coating has been detected for the sample with a 9 nm-thick spacer (Fig. 3(c)).

**Figure 4:**
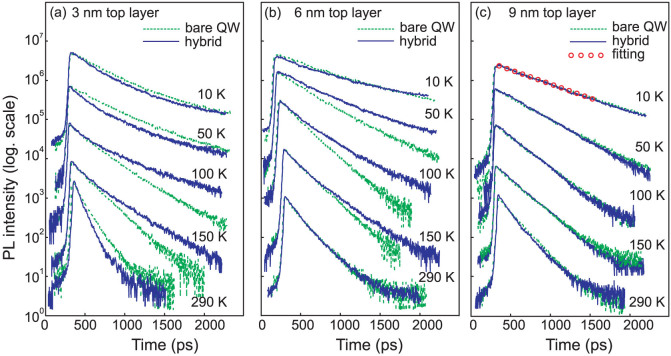
PL decay curves measured at different temperatures for the QW exciton peak position shown for coated (solid lines) and uncoated samples (dashed lines) for the spacer thickness of (a) 3 nm, (b) 6 nm, and (c) 9 nm. Temperature is indicated for each curve. An example of fitting using single exponential decay law is shown by open circles in (c) for the PL decay curve measured at 10 K.

**Figure 5:**
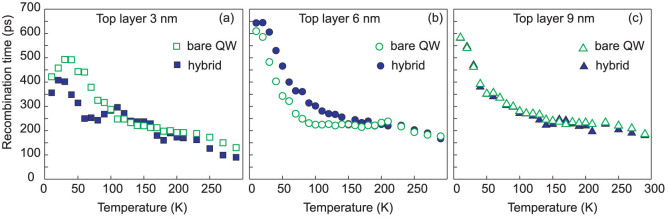
Extracted recombination time of the QW exciton shown as a function of temperature for the hybrids (solid signes) and for the uncoated samples (open signes) with the top layer thickness of (a) 3 nm, (b) 6 nm, and (c) 9 nm.

PL recombination time has been studied depending on the cap layer thickness in order to investigate a possibility of NRET between the QW exciton and the ZnO NCs. Fig. 4 shows typical examples of the PL decay curves taken at the exciton peak energy at several temperatures between 10 and 290 K. The results are compared for the bare QW samples (dashed lines) and after coating by the ZnO NC film (solid lines). Fig. 5 shows the PL recombination time τ of the QW exciton extracted from the experimental data by fitting using a single exponential decay law *I = I_0_exp(-t/τ)* as exemplified by open circles in Fig. 4(c) for one PL decay curve.

## Discussion

The following observations related to the QW exciton dynamic behavior can be pointed out: (i) In the case of 3 nm thick cap layer, the PL decay is faster in the hybrid for lower temperatures of 5–90 K, however a longer component of the recombination time start to be pronounced at ~100–200 K. Then again, between 200 and 290 K, the PL decay rate is increasing (Fig. 4(a)) and consequently, the QW exciton lifetime becomes shorter for the coated sample, see Fig. 5(a). (ii) For the hybrid fabricated using a structure having the cap layer of 6 nm, the QW exciton lifetime is longer compared to the bare QW structure in the temperature range of 30–180 K as shown in Fig. 4(b) and Fig. 5(b). (iii) There is no difference in exciton recombination between the hybrid and uncoated structures for the sample grown with the thickest spacer as seen in both Fig. 4(c) and Fig. 5(c).

We can conclude that there is no noticeable influence of the ZnO NCs film on the exciton dynamic in the sample with 9 nm cap layer, while there is a clear effect of the coating on the exciton temporal behavior in hybrids with thinner spacer. However, the tendency is opposite in two cases, i.e. for samples with 3 and 6 nm cap layer thickness. Thus, we suggest that there are several possible factors having opposite impact on the QW exciton recombination.

Let's consider the impact of NRET on exciton dynamics in our samples. The dependence of NRET on the distance *d* between donor and acceptor components of the hybrid structure is determined by its configuration and differs for layers, QW and NCs. The evaluation of the energy transfer rate in the weak-coupling regime from the QW to the overlayer material, which can be organic or inorganic semiconductor nanostructures was described by Agranovich et al.^9^ The rate of NRET, *k_ET_*, caused by dipole-dipole interaction in hybrid structures can be expressed as *k_ET_* ∝ *d*^−*n*^, where *n* depends on the dimensionality of the array of dipoles and can vary from 6 for NRET between two point-like molecules (0D-0D) and 2 for two thin layers or QWs (2D-2D)^10^. NRET in hybrids is an additional channel of non-radiative recombination, thus decreasing the lifetime of the excitons in QW. For the bare QW structures the recombination rate *k_QW_* has contribution of radiative and non-radiative recombination components: *k_QW_* = *k_r_* + *k_nr_*. For the hybrid structures assuming only one additional non-radiative recombination mechanism (i.e. NRET) it can be written: *k_H_* = *k_r_* + *k_nr_* + *k_ET_* and, thus, NRET can be determined from the measured exciton recombination rates in the hybrid and in the bare QW as *k_ET_* = *k_H_* − *k_QW_*^5^,^11^. However, there can be other recombination mechanisms in hybrids compared to the bare QW samples as we discuss below.

At temperatures of 5–40 K the QW excitons are localized on the potential fluctuations, while the exciton movement in ZnO NCs is limited even at elevated temperatures by the particle size of ~20–30 nm taking into account a diffusion length of 200 nm^12^. Consequently, at low temperatures the dimensionality of two dipoles is close to 0D and, thus, the NRET effect is significantly suppressed. With increasing *T* the degree of exciton localization decreases, hence the exciton becomes a 2D-particle. Accordingly, the NRET rate is higher at 50–100 K with a further reduction at elevated temperatures, when the wave vector of free excitons is rather large for efficient dipolar coupling as was previously reported for hybrids with organic acceptor layer^4^. Thus, it is likely that NRET can be responsible for the reduction of the recombination time at T < 100 K observed in the hybrid with the thinnest cap layer of 3 nm (Fig 5(a)). In the hybrids with the larger distance *d* (6 or 9 nm) the NRET rate is reduced by at least one order of magnitude and can be neglected.

**Figure 6:**
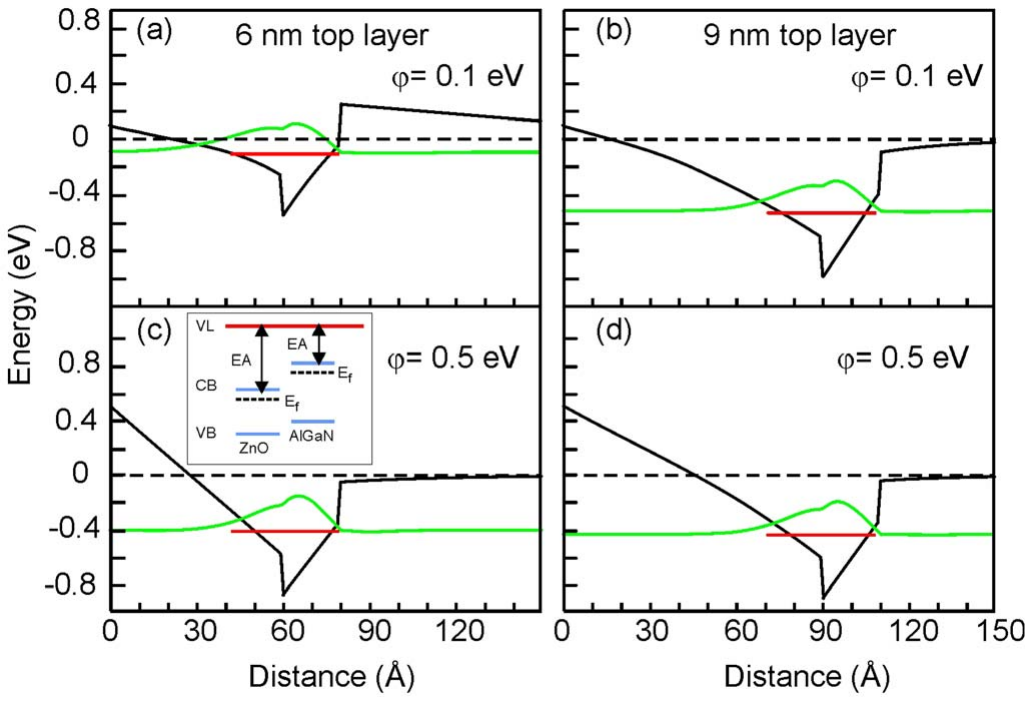
The conduction band profile calculated for the QW structures with different top barrier thickness of 6 nm (a, c) and 9 nm (b, d) are shown for two surface potentials: φ = 0.1 eV (a, b) and 0.5 eV (c, d). The occupied energy level of electron and its wavefunction are also shown for each case. The insert in (c) shows schematic drawing of the conduction band (CB), valence band (VB), the electron affinity (EA), the vacuum level (VL) and the Fermi energy (E_f_) for n-type ZnO and AlGaN.

Besides NRET we suggest to consider the surface potential effect on recombination times in hybrids. To explain the increase of the QW exciton lifetime at 20–200 K in the hybrid with *d* = 6 nm layer (see Fig. 4 (b) and Fig 5 (b)) and at 100–200 K in the hybrid with d = 3 nm (can be seen in Fig. 4(a) for T = 100 and 150 K), we assume that the surface potential in the QW structures can be changed in vicinity of the ZnO nanoparticles. We can estimate the effect of the surface barrier on the QW exciton using a self-consistent solution of the Schrödinger and Poisson equations^13^. Fig. 6 illustrates the band profiles for two QW structures with the top barrier thickness of 6 and 9 nm, respectively. Such consideration has shown that, if the cap layer is thin (3 or 6 nm), then the electron levels in QW are pushed up (~18 meV) for the surface potential of 0.5 eV compared to φ = 0.1 eV. On the other hand, the influence of the surface potential is negligible for the QW structure with a 9 nm-thick cap layer. Clearly, the potential gradient is stronger for the region close to the surface. Thus, for the samples with 3 and 6 nm cap layers a higher surface barrier will increase the carriers confinement in QW and at the same time will reduce an electron–hole overlap and hence oscillator strength due to an additional space separation of charges. We note here that if we consider a ZnO/AlGaN heterojunction (which is not a case in our hybrids), the potential barrier for the conduction band will be higher at the interface. A quantum confined Stark effect is stronger for wider wells^14^ and, thus, for excitons localized by the QW width fluctuations. Though the effect is small for 2 nm thin QW, it can explain a slower PL decay time observed at elevated temperatures in the hybrids with 3 and 6 nm cap layer as the interplay between recombination of excitons with different oscillator strength. At T>200 K, which corresponds to the localization energy of ~17 meV, all the QW excitons will be thermalized. Thus, the recombination time τ in QW will be determined by the dynamics of free excitons that are less sensitive to the potential profile. This is in agreement with our experimental results obtained at 200–290 K for the structure with *d* = 6 nm, when the QW exciton lifetime is the same for the coated and uncoated sample (Fig. 5(b)). Also, the thermal behavior of the recombination time observed for the hybrid with *d* = 3 nm can be understood in terms of at least two different mechanisms: (i) NRET, which results in decrease of τ (the effect is stronger between 50–100 K), and (ii) the variation of the surface potential in hybrids, which can contribute with a slow τ at temperatures up to 200 K. Other factors influencing the exciton lifetime in III-nitrides such as variation of stress, carrier density etc.^15^,^16^ can likely be neglected, since no effect was observed for the reference sample with a 9 nm-thick cap layer.

In summary, we have studied ZnO NCs/AlGaN/GaN hybrid structures designed to utilize NRET. Dynamic properties of the QW exciton in hybrids compared to the bare QW structures were studied depending on the cap layer thickness. We have found that the QW exciton lifetime decreases for hybrids compared to the bare QW structures with the thinnest (3 nm) cap layer at low temperatures of 5–90 K and above 200 K, while the QW PL decay became slower up to 200 K in the coated structure with the spacer thickness of 6 nm. No difference in the thermal behavior of the QW exciton lifetime was found between coated and uncoated structure with a 9 nm-thick cap layer. An increase of surface potential barrier is suggested as an additional mechanism, besides NRET, affecting QW exciton lifetime in hybrids.

## Methods

Three AlGaN/GaN/AlGaN QW structures were grown on sapphire substrates with a 1 µm thick GaN buffer layer by metal organic chemical vapor deposition (MOCVD) method using trimethyl gallium, trimethyl aluminum and ammonia as precursors. The growth temperature was 1050°C. The thickness of the GaN QW was ~2 nm for all three samples, while the thickness of the AlGaN cap layer (spacer) was 3, 6 and 9 nm, respectively. The Al composition in the AlGaN alloy was adjusted to ~16%. For fabrication of the hybrid structures, we have used colloidal ZnO NCs as an energy acceptor material. The powder of ZnO NCs was purchased from Sigma-Aldrich. An average diameter of particles was 30 nm. The ZnO NCs were dispersed in ethanol with a concentration of ~3.3 Vol.% and then spin-coated on the top of QW structures. TEM measurements were performed with a high resolution FEI Tecnai G2 200 keV FEG instrument, SEM has been done with a FEG cathode LEO 1550 Gemini scanning electron microscope. For PL measurements we have used the third harmonics (λ_e_ = 266 nm) from a Ti:sapphire femtosecond pulsed laser as an excitation source. The laser frequency was 75 MHz. TRPL was performed with a Hamamatsu synchroscan streak camera having a temporal resolution of ~ 20 ps. The samples were placed in a liquid helium cooled cryostat providing temperatures in the range of 5–300 K.

The band profile of the QW structures have been calculated using a self-consistent solution of the Schrödinger and Poisson equations^13^ with the same material parameters for AlGaN and GaN as used in Ref. 17. Calculations were performed for structures having *d* = 3, 6 and 9 nm for different surface potentials between 0.1 and 0.5 eV. Occupied levels of electron in the ground state together with an envelope functions have also been calculated. The polarization of 0.028 and 0.069 C/m^2^ has been used for the GaN QW and for AlGaN barrier, respectively.

## Author Contributions

G.P. designed the research idea. C.H. contributed to calculations and discussion of the paper. M.F. contributed to experimental measurements. H.A. contributed to sample growth and discussion of the paper. G.P. and M.F. contributed to writing the paper. All authors reviewed the manuscript.
